# Frequency Following Responses to Electric Cochlear Stimulation in an Animal Model

**DOI:** 10.1007/s10162-025-00992-3

**Published:** 2025-05-21

**Authors:** Matthew L. Richardson, Robert P. Carlyon, Harrison W. Lin, John C. Middlebrooks

**Affiliations:** 1 Department of Otolaryngology, Center for Hearing Research, University of California at Irvine, Irvine, CA USA; 2 Departments of Neurobiology & Behavior, Cognitive Sciences, Biomedical Engineering, University of California at Irvine, Irvine, CA USA; 3 Cambridge Hearing Group, MRC Cognition & Brain Sciences Unit, University of Cambridge, Cambridge, UK

**Keywords:** Frequency following response, Cochlear implant, Cat, Temporal pitch

## Abstract

**Purpose:**

Present-day cochlear-implant (CI) users can achieve high levels of speech reception in quiet surroundings. Nevertheless, sensitivity to the temporal pitch of sounds is limited, which contributes to deficits in speech reception amid multiple talkers and in appreciation of musical melodies. Short-term, invasive neurophysiological studies in animals have demonstrated limitations in neural phase locking in the tonotopic range of the auditory pathway that is activated by CIs. It remains an open question, however, whether those neural limitations can account for perceptual deficits in those animal species, let alone in human CI users. For that reason, we have evaluated non-invasive recordings of phase locking from cats chronically implanted with a CI.

**Methods:**

Ten deafened cats (eight female) were implanted with an animal version of a clinical CI array. The electrically evoked frequency following response (eFFRs) was recorded from the scalps of sedated animals at ≥ 10 weeks post-implantation. Stimuli consisted of constant-amplitude electrical pulse trains at rates from ~ 40 to 640 pulses per second.

**Results:**

Recordings of the eFFR demonstrated robust responses synchronized to electrical pulse trains across all stimulus rates. Analyses of eFFR amplitude and phase transfer functions confirmed that the eFFR, as with its normal-hearing counterpart, originates from multiple subcortical and cortical generators. The slopes of segments of eFFR phase transfer functions revealed stimulus-to-response latencies of generators that dominated the eFFR across restricted ranges of pulse rates. Those rate ranges must coincide with the limits of phase locking by putative generators at successive levels of the auditory neuroaxis and could inform our understanding of the limits to perceptual temporal acuity.

**Conclusion:**

The eFFR demonstrated here in an animal model provides a valuable non-invasive measure of temporal processing in electric cochlear stimulation that can be related to ongoing perceptual measures in the same animals and is well-suited to evaluate novel modes of auditory prosthesis for enhancing temporal acuity.

## Introduction

Present-day cochlear-implant (CI) users receive speech cues primarily by constant-rate electrical pulse trains that are modulated by the temporal envelopes of band-pass-filtered auditory signals. Although the slowly varying envelopes can provide reasonably good speech reception in quiet surroundings, most CI users have limited access to rapidly varying aspects of sound, including the cycle-by-cycle temporal fine structure (TFS) of the fundamental frequency (F0) of the human voice or other pitch-evoking sounds. For example, CI users can utilize electric TFS to rank the pitches of unmodulated pulse trains that vary in pulse rate, but performance varies widely across users and generally diminishes for rates greater than 300 pulses per second (pps) [1–3]. By contrast, it is widely thought that normal-hearing listeners employ acoustic TFS to discriminate the frequencies of pure tones at least up to 2000 Hz [4]. Although stimulation strategies have been developed to enhance the encoding of F0 periodicity, those strategies have afforded only modest benefits to CI users’ pitch and speech performance outcomes [5]. The limited temporal acuity by CI users contributes in large part to difficulties in common listening situations, such as identifying talkers amid competing sounds [6], appreciating music [7], and interpreting prosody cues (i.e., pitch contours) in speech [8].

Periodicity is encoded throughout the auditory pathway by activity of neurons that discharge synchronously to a particular phase of the stimulus. Single- and multi-unit recordings of phase-locked activity in experimental animals have provided valuable insights into why temporal acuity is limited with CI stimulation. Part of the problem could be neurodegenerative effects that result from long-term deafness. For example, auditory deprivation in chronically deafened animals was associated with degraded stimulus-synchronized spiking activity of neurons in the inferior colliculus [9–13] and primary auditory cortex [14, 15], which in some cases could be partially reversed by restoring hearing with a CI. Another possible explanation for poor TFS sensitivity is that conventional CIs stimulate only basal-to-middle cochlear turns, whereas an animal study has demonstrated superior transmission of TFS when apical cochlear fibers are selectively stimulated with a penetrating auditory-nerve electrode [16]. Nevertheless, it remains unknown how those neural limitations might account for perceptual deficits in the same animal species, let alone in human CI users.

The frequency following response (FFR) is the far-field potential of phase-locked activity that can be recorded non-invasively at the scalp. Characteristics of the FFR in normal-hearing humans have been associated with the fidelity of F0 encoding relevant to pitch perception (e.g., [17–19]). We recently showed that the FFR in normal hearing cats could be elicited by acoustic pulse trains composed of high-frequency, band-pass harmonic complexes, a stimulus that resembles the TFS of CI pulse trains [20]. Robust FFR was recorded over a range of stimulus rates of ~ 100 to 800 pps. Moreover, cats could discriminate changes in rate over that same range in accompanying psychophysical measures. Following on that work, the present study aimed to evaluate the electrically evoked FFR (eFFR) in cats that were implanted with an animal version of a clinical CI. The benefit of that animal model is that, unlike human studies, we have experimental control over factors that might influence temporal acuity (e.g., etiology and duration of deafness). Unlike the invasive recordings of single- and multi-unit activity, the eFFR permits longitudinal measures of the cat’s neural phase locking that can be readily related to the same animals’ perception and are also comparable to eFFR of human CI users.

Importantly, the scalp-recorded FFR reflects the aggregated phase locking of multiple subcortical and cortical auditory generators. The contributions of multiple generators have been recently highlighted by human FFR studies (e.g., [21]) but were originally reported by studies of normal-hearing animals [22–24]. For example, Gardi and colleagues [24] demonstrated that minima and maxima in the cat FFR amplitude transfer function can be modeled by the sum of multiple sinusoids, each having delays corresponding to successive nuclei along the neuroaxis. Kuwada and colleagues [25] extended that work, in rabbits, by demonstrating correspondences of phase characteristics of the FFR with latencies recorded invasively from putative generators. That study also highlighted the dominance of longer, cortical-like, latencies at low stimulus frequencies compared to shorter, midbrain- or brainstem-like, latencies at higher frequencies. Those latency transitions presumably reflected the reduced phase-locking capacities at ascending levels of the auditory pathway.

Here, we recorded the eFFR from the scalps of deafened cats implanted with chronic CIs. We tested ranges of electric pulse rates that are relevant to temporal pitch perception in normal-hearing cats [20] and to spiking neuronal activity in the midbrain of acutely implanted cats [16]. We addressed several questions that assessed the utility of the eFFR for probing neural transmission of TFS by electric cochlear stimulation. Can the periodic neural responses be effectively separated from contaminating electric stimulation artifact? Do analyses of eFFR amplitudes and latencies provide evidence that the eFFR—as with the normal-hearing FFR—originates from multiple neural generators? And most importantly, can the latencies of the eFFR across pulse rates provide insights into the maximum phase-locking capacities of putative neural generators along the electrically stimulated auditory pathway? The answers to these questions demonstrate the value of non-invasive eFFR for studying the neural basis of temporal pitch perception in the cat animal model of chronic auditory prosthesis. Future studies can combine the eFFR with cat psychophysics to evaluate novel interventions for enhancing temporal acuity by electric stimulation.

## Methods

### Animals

All methods and procedures were approved by the Institutional Animal Care and Use Committee at the University of California at Irvine in accordance with the NIH Animal Welfare Guidelines and are reported here following the ARRIVE (Animal Research: Reporting of the In Vivo Experiments) guidelines. Domestic shorthaired cats (*Felis catus*) were obtained from a breeding colony at the University of California at Davis. The animals were group-housed and given free access to dry food and water in the housing area.

Data are presented from ten cats (two male, eight female) that were chronically implanted with an animal version of a clinical CI electrode array (described in the following section). Three of the cats received only the CI array. An additional seven cats were implanted with a dual-electrode device that had the same CI array, supplemented by a single-shank electrode that was positioned in the auditory nerve as part of a separate ongoing study. The present study reports only data for stimulation by the CI arrays; there was no indication that the presence of the unstimulated single-shank devices influenced the responses to the CI arrays. On the day of implantation, the cats ranged in age from 10.2 to 79.1 months (median = 23.2 months), including six cats < 24 and four cats ≳36 months, and weighed from 2.8 to 4.7 kg (median = 3.1 kg).

Scalp recordings were obtained regularly at ~ 2–3-week intervals after implantation. Here, we focus on recordings generally taken on sessions nearest to 10 weeks after implantation, which was regarded as a benchmark for stability of chronic implantation. Exceptions to that included two CI-only cats, NU and MO. Those cats were implanted at an earlier stage of the study and were tested with a more limited eFFR stimulus set before we changed to the present protocol at their post-implantation weeks 18 and 24, respectively; their data from those weeks are presented here as we observed no functional changes in neural responses over those longer post-implantation durations (addressed in the “Results” section). Additionally, data for cat CL are from week 6 as that cat exhibited hardware failure of the implant device at week 8 but showed robust scalp-recorded responses prior to the failure. Any data reported other than at the ~ 10-week session are noted in the “Results” section.

### Deafening and Chronic Implantation

All cats were deafened bilaterally ~ 2 weeks prior to implantation according to a protocol that has been previously described [13, 26]. Briefly, each cat was sedated (see details below, *Scalp Recordings*) and auditory brainstem responses (ABR) were recorded in response to broad-band noise bursts, ≥ 10 ms in duration, at varying sound intensities to obtain thresholds of elicited neural responses; noise bursts were presented through a horn tweeter placed 20 cm to the left of the cat’s left pinna. An ototoxin was then administered (kanamycin; 300 mg/kg dissolved in saline; sub-cutaneous) and, after a delay of ~ 30 min, the diuretic ethacrynic acid was infused intravenously (1 mg/ml dissolved in saline) in doses of 10–20 mg at 10-min intervals until the ABR threshold was elevated beyond the maximum equipment level, typically > 70 dB above the initial threshold. For ~ 1 h thereafter, ABR responses were monitored for any recovery of hearing thresholds and subsequent doses of ethacrynic acid doses were given as needed; the total ethacrynic acid amount ranged from 10.7 to 22.6 mg/kg, median 15.2. In three of the nine cases, residual hearing was detected on the day of implantation ~ 2 weeks after the original deafening, and it was necessary to repeat the deafening procedure.

Each cat was implanted chronically with a CI consisting of an eight-channel lateral-wall research electrode array manufactured by Advanced Bionics, LLC (Valencia, CA). The electrodes were platinum half cylinders, each with a geometric surface area of 1.1 × 10^5^ µm^2^ and separated center to center by 0.75 mm. The electrical return electrode was a platinum cylinder, 1.5 mm in diameter and 1.6 mm long. The CI array and return electrode were each attached to flexible coiled cables that led to a multi-contact percutaneous connector.

The implantation procedure was conducted in aseptic conditions while the cats were anesthetized with isoflurane gas; anesthesia and vital signs were monitored by a university veterinarian. The tympanic bulla was first exposed and then opened with a carbide burr to visualize the round window. The electrode array was inserted through the round window as far as possible in the scala tympani so that the distal, most apical, electrode was about halfway around the basal turn. That apical electrode was 8.75 mm distal to the base of the array, which typically fell at or just deep to the round window margin. The electrode array was then secured in place by filling the bulla with acrylic dental cement. The return electrode was positioned deep in the neck musculature caudal to the bulla. The coiled cables from the array and return electrodes were led under the temporal muscle to the percutaneous connector where it was placed on the dorsal midline of the skull. The connector was set inside a protective stainless-steel cylinder, and both were fixed in place with skull screws and acrylic cement. The surgical incision was closed, and the cylinder was covered with a screw-on cap that shielded the connector.

The continuity of the CI device was measured by the electrode impedance during each recording session. The impedance was computed from the voltage needed to pass a ± 5-µA current of a 6-kHz sinusoidal probe, which had a period (166.7 µs), similar to that of the electrical stimulation biphasic pulses (164 µs). Impedances of the most apical CI electrodes of each cat ranged from 6 to 14 kOhm (median = 6.4 kOhm) for the recording sessions reported here.

### Electrical Stimulation and Scalp Recordings

Stimulus generation and data acquisition used System III hardware from Tucker-Davis Technologies (TDT; Alachua, FL) controlled by custom MATLAB software (The MathWorks, Natick, MA) on a Windows-based desktop computer. The electrical stimuli were generated by an optically isolated current-controlled IZ2H stimulator from TDT. The output sample rate was 24,414 samples/s. Scalp recordings were made with a TDT Medusa4Z amplifier also having a sample rate of 24,414 samples/s; the amplifier had an onboard analog filter with a bandpass of 0.3 to 10,986 Hz.

Electrical stimuli consisted of symmetrical biphasic pulses, 82-µs per phase in duration with no inter-phase gap (164 µs total duration). Pulses with cathodic- or anodic-leading polarity were alternated across trials for the purpose of reducing electric artifact after trial averaging. Stimulation was always delivered to the most apical CI electrode in the results reported here. That electrode had the tightest fit in the scala tympani so that it generally produced the lowest neural activation thresholds and it stimulated auditory-nerve fibers with the lowest characteristic frequencies accessible to the CI array, which potentially provided the greatest temporal acuity [16].

Stimuli for the eFFR recordings were 500-ms trains of electric pulses, fixed in current level, that varied across trials in the rate of pulses per second (pps). Trains were repeated at 1200-ms intervals, onset-to-onset, with the inter-train interval jittered by 5% intended to mitigate entrainment with intrinsic oscillatory activity. The pulse rates ranged from 43 to 642 pps and were restricted to integer divisors of the output sample rate. All cats were tested with a sample of 31 pulse rates with increments ranging from 5 to 32 pps. Pulse trains with fixed polarity of pulses were presented so that each rate was presented once in a random order, then again in a new order with the opposite polarity, and so on, until 40 repetitions were presented for each rate, including 20 trials each of the anodic- and cathodic-leading polarity. The recorded epoch for each trial included 100 ms before to 650 ms after the pulse-train onset.

Current levels for the eFFR stimuli were determined by measuring the growth of electrically evoked ABR (eABR) responses to single pulses at 1-dB increments of current. At higher current levels, an electromyographic (EMG) response often was elicited, sometimes accompanied by visible movement of the pinna, and was attributed to activation of the facial nerve [27]. The EMG was identified by marked changes in waveform morphology and a steep growth of amplitude, i.e., 10 to 100 times increases in magnitude over a 2-dB increase in current level. The eFFR stimulus level for each cat was set at a high point of the eABR growth function that was at least 2 dB below the lowest level at which the EMG was noted. Those eFFR current levels ranged from + 5.9 to + 10.2 dB (median = 8 dB, interquartile range = 2.3 dB) relative to each cat’s eABR threshold level at which a neural response could be detected compared to a no-stimulus condition.

Electrophysiological recordings began approximately 2 weeks after implantation (i.e., ~ 4 weeks after deafening). In each session, cats were sedated using a light level of anesthesia induced with an intramuscular injection of ketamine (25 mg/kg) and acepromazine (1 mg/kg). At those doses, the cats were typically areflexic with no spontaneous movement, although eye-blink or limb-withdrawal reflexes sometimes could be elicited at later times in the recording session. Supplemental doses of ketamine alone were given when necessary to maintain an immobile state.

Scalp potentials were obtained with subdermal electrodes consisting of hypodermic needles. The electrodes were rubbed with an abrasive cloth to remove any silicone coating, re-sterilized, and inserted through the skin. The reference electrode was placed on the midline of the head, rostral to the percutaneous connector and approximately 1–2 cm above the supraorbital margin of the eyes. One active electrode was placed over the left mastoid, ipsilateral to the CI, and a second active electrode was placed over the right mastoid, contralateral to the CI. The symmetric configuration of the two active-electrode channels on the head permitted a general characterization of responses at the two hemispheres, each having varying proximity to neural generators of the auditory pathway. A ground electrode was placed on the cat’s back, caudal to the scapulae. The recorded trial data were high-pass filtered at 3 Hz to eliminate DC voltage and then stored to the computer. Trial-averaged and filtered data were displayed for online monitoring. Note, because the recordings were referenced with respect to the midline scalp electrode, we reversed the polarity of all waveform data in the following eFFR analyses, which conforms with the more conventional practice of using the mastoid(s) as the reference site in electrophysiological recordings. That is, midline-positive waveform deflections are drawn as upward going and deflections from predominantly unilateral sources (e.g., the cochlear nucleus) appear opposite in sign when recorded on ipsi- versus contralateral channels.

### eFFR Data Analysis

All signal processing and statistical analyses were performed in MATLAB (version 2023b). The eFFR was analyzed in terms of the frequency-domain amplitude and phase values of synchronized responses at each pulse rate. Electric pulse artifacts pose a particular challenge to eFFR analysis because the periodic artifact has similar spectral characteristics to that of the FFR, and can superimpose on the ongoing neural responses, making it difficult to isolate from the waveform. To address these challenges, we adapted strategies previously used in human eFFR studies in which the trial waveforms were first submitted to a multi-step artifact reduction procedure [28–32]. That procedure is described and evaluated in the following “eFFR Artifact Reduction” section. 

After artifact reductions, the trial waveforms were sorted with respect to pulse rate and stimulus polarity. Segments from 0 to ~ 50 ms re pulse-train onset were excluded from the analysis to omit any onset-specific responses, yielding an ~ 450-ms waveform duration. Trial waveforms for each pulse rate were screened for excessive noise assessed by the variance computed over time. Outlier trials were excluded if the variance exceeded the 75th percentile of variances across trials by more than 1.5 times the interquartile range, which on average amounted to rejection of 4.2 ± 2% (± standard deviation) of trials. The remaining trial waveforms were reconstituted by pairwise averaging of trials with anodic- and cathodic-leading stimulus polarity; that procedure provided the cancellation of any residual opposite-phase artifact to facilitate trial-level statistical analysis. Averages were then formed from all trial waveforms of each pulse rate, referred to here as the full-length waveforms. Additionally, the waveforms were segmented into ~ 23-ms intervals as adjusted to the nearest integer multiple of each pulse-rate period. Averages formed from those segments are referred to as the folded waveforms. The folding interval was based on the 23.3-ms period of the lowest pulse rate, 43 pps. For example, the waveform for 152.6 pps was folded in time over intervals of 26.2 ms (i.e., its period of 6.55 ms × 4), which yielded 17 waveform segments across the 450-ms waveform duration. Across all pulse rates, folded waveforms were formed from 12 to 20 segments (median = 19). All illustrated waveforms in the results were bandpass filtered from 40 to 1500 Hz; the filter order was set to 1, which was doubled by the bandpass design and doubled again by use of the MATLAB zero-phase *filtfilt* function, resulting in a fourth-order filter.

The fast Fourier transform (FFT) was computed for the full-length and folded waveforms, and eFFR transfer functions were obtained by extracting the spectral amplitude and phase at each pulse-rate frequency (F0). Prior to the FFT, the waveforms were tapered by Hann-windowed onset/offset ramps having durations of 100 ms for the full-length waveforms and 10 ms for the folded waveforms. All waveforms then were zero-padded so that the FFT output had 0.5-Hz frequency bins. The analysis of eFFR amplitudes utilized the full-length waveform spectra because of the greater inherent frequency resolution as compared to the shorter folded waveforms. For evaluating the artifact reduction procedures, additional amplitude values were taken at 2 to 4 multiples of the F0; those higher harmonics generally showed greater sensitivity (i.e., change in magnitude) than the F0 to the presence of the highly non-sinusoidal artifact. The noise floor level was estimated for each amplitude value by averaging 12 adjacent spectral bins (6 on each side) with 2-Hz spacing between bins to match the original frequency resolution of the ~ 500-ms pulse trains. The analysis of eFFR phase utilized the folded waveform spectra because the additional averaging constituting those waveforms was found to provide smoother progressions of phase lag as function of the pulse rate.

#### A Delay-and-Add Model of Amplitude Transfer Function

The amplitude transfer functions typically contained local minima and maxima (“dips” and “peaks”) across the tested pulse rates. Those spectral amplitude features presumably reflected the frequency-dependent destructive and constructive sums of multiple neural generators recorded simultaneously at the scalp [24, 25, 33]. To test whether that sum-of-generators hypothesis could account for the present amplitude features by electric stimulation, we adapted a delay-and-add modeling approach that was developed initially for FFR in normal-hearing cats [24] and expanded upon for normal-hearing humans [33].

The periodic responses of scalp-recorded neural generators were simulated by sets of four sinusoids, 500-ms in duration, all equal in frequency to the F0 of each stimulus. For each F0, the four sinusoids were generated having relative delays corresponding to biologically plausible latencies of particular generators: cochlear nucleus (fixed at 1.2 ms), other early brainstem generators including superior olivary complex (2.0 to 4 ms), inferior colliculus (4 to 8 ms), and thalamic/cortical generators (8 to 24 ms); an initial version of the model used separate thalamocortical and cortical generators, but that yielded only qualitatively, not statistically improved results. The fixed cochlear-nucleus latency was based on the first response after pulse onset in the eFFR waveforms, which was prominent and consistent across cats. The ranges of latency for the three subsequent generators were based on published measures of electrically evoked first-spike latencies and group delays of spiking activity of the respective auditory nuclei [9, 10, 14, 16, 34–37]. Values of latency within the three ranges were tested exhaustively by summing the sinusoids with particular sets of latencies and computing the resulting amplitude transfer functions across the range of stimulus rates; to reduce the search space, candidate latencies were restricted within each range by increments of ~ 0.5, ~ 0.5, and ~ 1.25 ms, respectively, for the brainstem, inferior-colliculus, and thalamic/cortical generators. In addition, following Tichko and Skoe [33], we scaled the relative magnitudes of the sinusoids to account for differences in proximity of generators to the recording electrodes (e.g., larger cortical than brainstem responses) as well as anatomical or physiological variations among cats. Exploratory testing revealed generally good model fits to empirical amplitudes with the following scalars: cochlear nucleus (fixed at 1), brainstem (fixed at 1), inferior colliculus (varied from 2 to5), and thalamic/cortical (varied from 5 to 10). Also, to account for declining phase locking at higher levels of the auditory neuroaxis, the inferior colliculus and thalamic/cortical generators were linearly tapered from full to zero amplitude, starting from the respective cut-off frequencies of 250 and 90 Hz and extending an octave above those cut-offs. The various model transfer functions were compared with empirical transfer functions by finding the sets of latencies and generator scalars that minimized the frequency differences of peaks and dips and overall amplitude differences between the modeled and empirical transfer functions.

#### Group Delays Computed from Phase Transfer Functions

The phase lags of the empirical transfer functions were unwrapped by accumulating phases across increasing pulse rates, adding 2π radians wherever there was a difference greater than π between phases of adjacent rates (*unwrap* function in MATLAB). The eFFR response latencies were estimated by the group delays, which are given by the slopes of portions of phase transfer functions that are linearly related to pulse rate [38]. We generally observed systematic transitions from steeper (e.g., longer-latency) to shallower (e.g., shorter-latency) phase slopes with increasing pulse rates (cf. [25, 39]). We employed two methods to evaluate slopes of transfer functions across limited ranges of pulse rate: piecewise linear regression and 3-point running group delay. The *piecewise regression* method partitioned the pulse rates into 5 linearly fitted segments that taken together minimized the sum of squared errors among all possible combinations of 5 ranges of pulse rates. Here, the 5 segments were fit to all statistically significant cumulative phase values (a maximum of 31), with each segment consisting of a minimum of three or more pulse rates. Only 4 segments were used in some cases that had 20 or fewer significant pulse rates. Note, the number of segments was based on fitting considerations and assumed neither the number of generators contributing to the scalp-recorded responses nor the independence of generators in the group-delay latency estimates. After fitting the 5 (or 4) segments, adjacent segments that did not differ significantly in slope (*p* > 0.05, Analysis of Covariance) were fused into a single segment. The result of each piecewise regression analysis was a set of 2 to 5 line segments (median = 5, 25th–75th percentile = 4–5), each segment restricted to a range of pulse rates and yielding a group delay. The *3-point running group delay* method provided a more continuous estimate of latency as a function of pulse rate. Here, group delays were computed for successive sets of three consecutive pulse rates, so that linearly fitting started with the lowest three rates ranking 1–3, followed by ranks 2–4, then 3–5, and so on until the highest three rates. Given up to 31 pulse rates, that analysis could yield a sequence of as many as 29 group delays. Those group delays then were linearly interpolated to provide 1-pps resolution.

Both methods for analysis of phase slopes had two additional constraints. First, to maintain relatively localized estimates of latency, fitting did not occur over gaps in pulse rate wider than 60 pps (80 pps in cases of < 20 significant pulse rates). Second, fitting was applied only to cumulative phases that increased monotonically, while allowing only for phase reductions ≥ -π/20 between adjacent pulse rates. That tolerance accounted for possible noise in the phase measurement and was based on the 95th percentile of residual errors taken from fitted segments restricted to purely monotonic cumulative phases, aggregated across all cats and recording sessions.

#### Statistics

The statistical significance of the eFFR for individual cats at each pulse rate was obtained by the Rayleigh test for non-uniformity of phase values across trials, assessed at the level of *p* < 0.01 [40]. Group-level analyses were based on a repeated measures experimental design with each animal as the experimental unit. Repeated measures analysis of variance (RMANOVA) tested for main effects and interactions for the factors of pulse rate, recording channel (ipsilateral vs. contralateral), and phase-lag analysis method (i.e., piecewise linear regression vs. 3-point running group delay). Significance was assessed at the level of *p* < 0.05 with the Greenhouse–Geisser (G–G) correction applied for non-sphericity, although the original degrees of freedom are reported. Post hoc pairwise comparisons used the non-parametric two-tailed Wilcoxon signed-rank test. Whenever appropriate, a 5% false discovery rate (FDR) correction was applied to account for multiple comparisons [41].

### eFFR Artifact Reduction

**Fig. 1:**
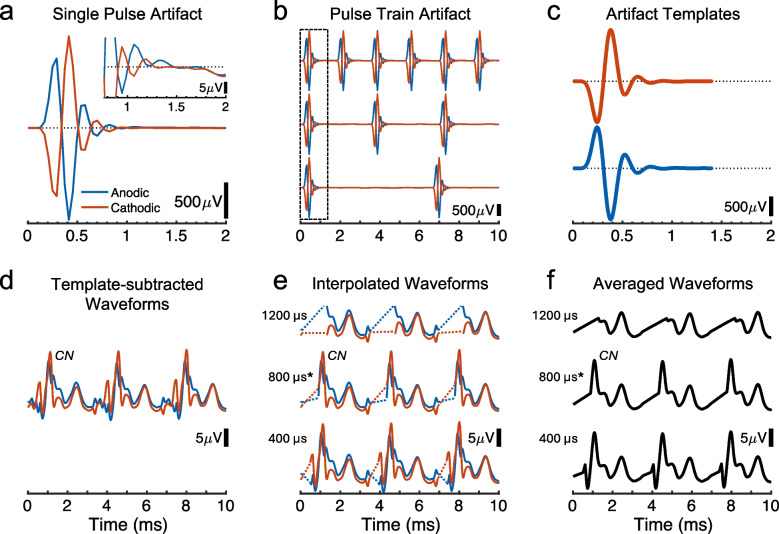
eFFR Artifact reduction procedures. **a** An example of artifact by a single biphasic pulse, from 0 to 2 ms re pulse onset, for both the anodic- (blue) and cathodic-leading (red) pulses, 82 μs/phase. The inset shows a higher-gain view (×100) of the artifact from 0.8 to 2 ms. **b** Examples of artifact recorded for eFFR pulse trains for three experimental pulse rates. The dashed box indicates the time window of 0 to 1.4 ms used to construct the artifact template. **c** Examples of the artifact templates for anodic- and cathodic-leading pulses. **d** The recorded eFFR waveform for three stimulus cycles of a single pulse rate (290 pps) after the template was subtracted from each pulse artifact. Note the ×100 higher gain compared to panels a-–c. The CN label indicates the earliest neural response peak identified, attributed to the cochlear nucleus. **e** The same eFFR waveform as panel d, after blanking and interpolating over three possible durations relative to pulse onset. The asterisk after 800 μs indicates the blanking length that was selected for routine artifact reduction. **f** The same eFFR waveforms as panel e, after averaging across the two stimulus polarities

The eFFR trial waveforms for each recording channel were submitted to an artifact reduction procedure that consisted of the following steps: (1) template subtraction, (2) blanking and linear interpolation, and (3) averaging across trials that alternated in stimulus polarity. Figure 1a shows that the electric pulse artifacts had maximum amplitudes from 0 to ~ 600 µs after pulse onset, followed by amplifier-induced ringing with diminishing amplitudes up to ~ 1400 µs; see × 100 higher gain in the Fig. 1a inset. Blue and red lines indicate anodic- and cathodic-leading pulse polarities, respectively. Figure 1b shows examples of the artifact for segments of eFFR pulse trains and Fig. 1c–f shows the artifact reduction steps.

In the template method, a model of the artifact was constructed and then the model was subtracted from the recorded waveform. As a first step, the recorded waveforms were up sampled by 10 times the original sample rate. That served to reduce any aliasing effects and to homogenize the shape of the artifact, thereby providing both a more accurate template model and greater precision in aligning the templates to the recorded waveforms prior to subtraction. Separate templates of single electric pulses were formed for each of the anodic- and cathodic-leading stimulus polarities. As depicted by the dashed rectangle in Fig. 1b, the templates were derived from the first pulse of each of the pulse trains for a given polarity, regardless of pulse rate, and then averaged together. The template interval started at 0 µs and ended at 1400 µs re the pulse-train onset. Importantly, the template interval for the first pulse in each train was absent of any ongoing neural responses to preceding pulses because of the 700-ms no-stimulus interval between subsequent trials. Nevertheless, the later portion of the template interval could contain a short-latency neural response to the first pulse, occurring as early as 1 ms after the pulse onset. To isolate that neural response from the templates, we exploited the opposite phase polarities of the anodic and cathodic templates, which could be averaged together to yield primarily the equal-phase neural activity. That average waveform trace was then subtracted from the separate anodic and cathodic templates. That yielded the single-pulse templates for the two polarities (see Fig. 1c). Those were concatenated into a sequence of templates that were matched in time to recorded pulse artifacts for each pulse rate. Time alignments were achieved by cross correlating the single-pulse template and FFR waveforms and computing the peak lag indices; that procedure accounted for occasional stimulating or recording asynchrony, which was never more than ± 41 µs (i.e., ± 1 sampling point). Last, to account for any amplitude fluctuations over the recording duration, each single-pulse template in the sequence was scaled to have the same peak-to-peak amplitude as its assigned pulse artifact in the recorded waveform.

The artifact template sequences were subtracted from the respective trial waveforms for each pulse rate and polarity. Figure 1d depicts a waveform segment after template subtraction and demonstrates that pulse artifacts could be effectively reduced, often to magnitudes near or below the neural response amplitude; note the × 100 difference in amplitude scales between Fig. 1a–c and d. We found that the first neural response following the largest portion of pulse artifact could be attributed to the cochlear nucleus (CN), which occurred at ~ 1.2 ms after pulse onset. For our purposes, the time points before the CN response were considered irrelevant; that included the far-field compound action potential from the auditory nerve, which in cat has electrically evoked latencies of ~ 500 µs and could be difficult to isolate from artifact [27, 42]. In Fig. 1d, the positive-going CN response is distinguished by having similar phase characteristics regardless of stimulus polarities; as described in the results, that response also shows longer latencies and reduced amplitudes with increasing stimulus rate, which is expected for a neural response but not an electric artifact. Nevertheless, remaining artifact effects could often be detected at time points before the CN response. For that reason, a segment of the template-subtracted intervals was blanked, and the data were linearly interpolated across the blanked samples. Figure 1e shows the blanking and interpolation procedure for 3 blanking lengths (dashed lines), which always started at 50 µs after the pulse onset. We evaluated a range of blanking lengths from 200 to 1600 µs to find the minimal duration necessary that showed evidence of reducing the influence by residual artifact, that yielded amplitude and phase responses characteristic of neural activity, and that was stable among adjacent blanking lengths, indicating low volatility to small manipulations. Figure 1f shows the averaging across stimulus polarity for the 3 example blanking lengths, which almost entirely eliminated residual effects of the opposite-phase artifacts. Asterisks indicate the 800-µs blanking length chosen for the main analysis as described in the following section.

**Fig. 2:**
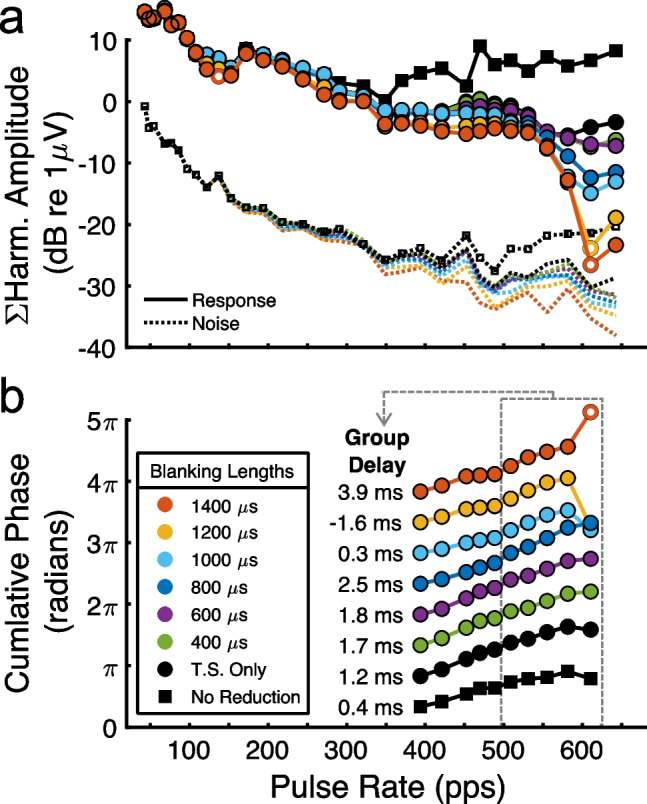
Evaluation of the artifact reduction procedures in one representative cat. Colors indicate the separate artifact removal conditions, including no artifact reduction (black squares, “No Reduction”), template subtraction alone (black circles, “T.S. Only”), and various blanking lengths applied after template subtraction. **a** Solid lines with symbols depict the spectral amplitudes summed for each pulse-rate F0 and its harmonics 2-–4 for the artifact reduction conditions. Dashed lines are the noise levels estimated at each pulse rate from the spectral bins adjacent to the F0 or harmonics frequencies and summed. **b** Cumulative phase values for pulse rates greater than 350 pps for each artifact reduction condition. The vertical positions of the various curves are arbitrary. The dashed-line box indicates the range of pulse rates (>500 pps) over which the slopes of the phase progressions yielded group delays that were used to evaluate the effects of artifact reduction in each condition

Figure 2 shows in one example cat a representative pattern of amplitude and phase responses that resulted from various conditions of artifact reduction. Compared to no artifact reduction (black squares, “Artifact”), Fig. 2a shows that the initial step of template subtraction (black circles, “T.S. Only”) markedly reduced both the response (solid lines) and noise (dotted lines) amplitudes. Those amplitude reductions were largest at pulse rates above ~ 350 pps, presumably due to the increased number of the artifacts being subtracted per unit of time. By examining the highest pulse rate, 642 pps, one can see that applying interpolation over short blanking lengths (400–600 µs) reduced the amplitude only slightly compared to template subtraction alone. There was another intermediate amplitude reduction for lengths of 800 to 1000 µs, but at yet longer lengths the responses were highly diminished or became non-significant (open symbols), likely because of excessive blanking that could extend over 90% of the stimulus period at the highest pulse rates.

Figure 2b shows the cumulative phase lags over a range of the higher pulses rates, > 350 pps; the vertical positions of curves are arbitrary. We computed group-delay latencies from the slopes of cumulative phase only for the highest of those rates (> 500 pps; see dashed-line box), which were expected to show the strongest influence from the artifact. Indeed, the group delay computed prior to artifact reductions was 0.4 ms, corresponding with the latency of maximum amplitudes of the artifact (see Fig. 2a). By reducing the influence of the artifact, however, template subtraction and interpolation over increasing blanking lengths tended to produce longer-latency group delays, here up to 2.5 ms for a blanking length of 800 µs, consistent with response latencies of the auditory brainstem. At longer blanking lengths, the group delays began to decrease again, or phase progressions became unstable by including non-significant responses; note the aberrant phase values at the highest pulse rate for blanking lengths > 1000 µs, indicative of excessive blanking. Based on those amplitude and phase results, which were comparable across cats and recording channels, a blanking length of 800 µs, extending from 50 to 850 µs after pulse onset, was applied to all eFFR waveforms. That blanking length was ~ 3 to ~ 51% of the inter-pulse interval for the lowest to highest tested pulse rate, 43 to 642 pps, respectively.

## Results

### eFFR Waveform Features

**Fig. 3:**
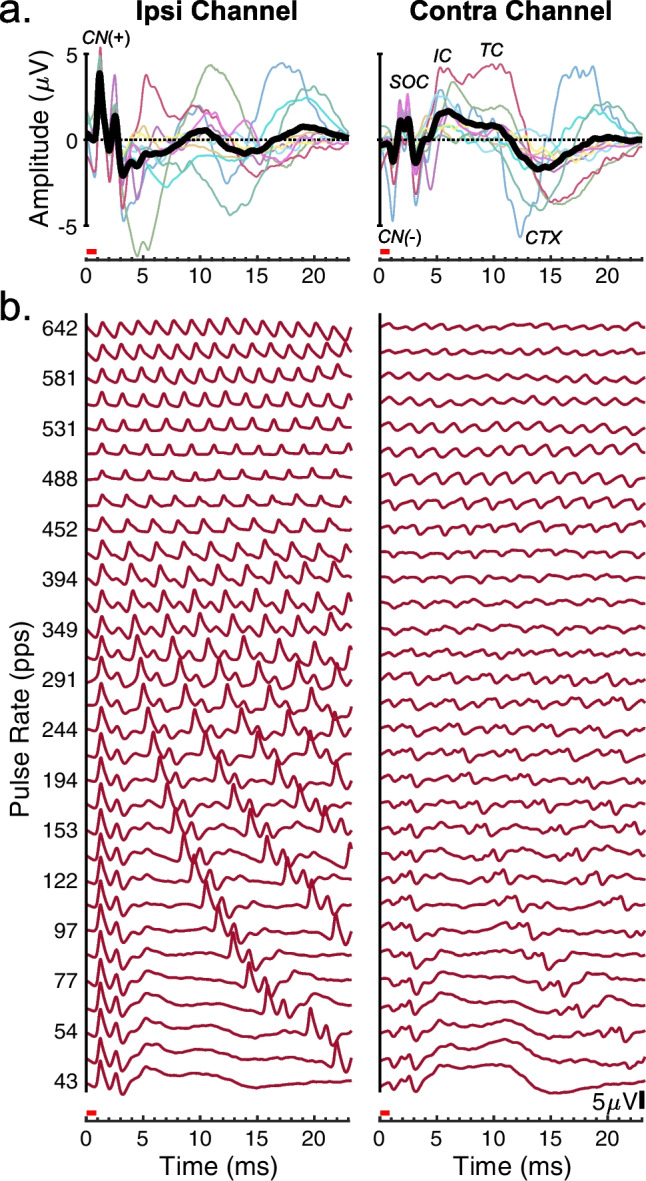
eFFR Waveforms, passband 40 to 1500 Hz. **a** Folded eFFR waveforms for the 43-pps pulse rate for individual cats (thin lines, various colors) and the grand mean (thick black lines) across cats. Ipsi and contra are the hemispheres of the recording electrodes with respect to the hemisphere of the CI. Abbreviations indicate responses attributed to various eFFR generators: CN, cochlear nucleus; SOC, superior olivary complex and other brainstem structures; IC, inferior colliculus; TC, thalamocortical; CTX, cortex. **b** Example of the folded eFFR waveforms across all experimental pulse rates for Cat BR on post-implantation week 10. The red bars above the horizontal axes indicate the time range of artifact removal by blanking

The eFFR recordings were evaluated for probing the transmission of temporal fine structure through ascending levels of the auditory system. It has been demonstrated that the normal-hearing FFR reflects the phase-locked activity from the auditory nerve combined with that of various auditory brainstem, midbrain, and thalamocortical nuclei (animal: [24, 25, 39], human: [21]). Here, we initially consider the temporal features of the eFFR waveform at low pulse rates, which can provide insight to the possible neural generators contributing to the present recordings. Figure 3a shows the eFFR waveforms for the 43-pps pulse rate recorded with ipsilateral (left panel) and contralateral (right) recording-electrode placements; throughout, ipsilateral and contralateral are given with respect to the side of the stimulating electrode. The thin lines show waveforms from individual cats and the thicker black lines show the grand means across cats. As described in the “Methods” section, the waveforms were folded in time over the period of the illustrated pulse rate (43 pps). The folded eFFR waveforms at that pulse rate had a relatively long inter-pulse interval that contained less overlapping activity by sequential pulses than at higher rates. All waveforms in Fig. 3 are bandpass filtered from 40 to 1500 Hz and are shown from 0 to 23 ms re pulse onset.

The grand mean eFFR waveforms over all cats at 43 pps contained response features that could be attributed to putative neural generators of the auditory pathway. The first deflection occurring at a latency of ~ 1.2 ms was regarded as an initial cochlear-nucleus response (CN, i.e., corresponding to ABR wave II in normal-hearing cats: [27, 43, 44]). The compound action potential of the auditory nerve occurring prior to 1 ms was lost in the period of blanking and interpolation shown by the red bar. Note, the CN had opposite response polarities between the channels, positive-going in the ipsilateral (see “CN (+)” in Fig. 3a) and negative-going in the contralateral channel (see “CN (-)”), possibly due to the local dipole orientation of the CN potential relative to the two recording configurations. The CN was followed by positive- and negative-going peaks, from ~ 1.5 to 4 ms after pulse onset. We attribute those features to the superior olivary complex (SOC) with possible contributions of other lower-brainstem structures such as additional cochlear nucleus or lateral lemniscus activity [34]. For simplicity, we denote those peaks as “SOC” in the remaining text.

Following the lower-brainstem responses, there was a positive deflection centered between ~ 4 and 8 ms (see Contra Channel) corresponding to the range of latencies of electrically evoked spike activity in the inferior colliculus (denoted “IC”, [16, 36]). The IC peaks are followed by another positive deflection between 8 and 12 ms, which we attribute to thalamocortical activity of the medial geniculate body and its excitatory post-synaptic potentials in the primary auditory cortex (denoted “TC”), and then by broad negative peaks occurring at latencies > 12 ms, which we attribute to spiking activity in primary auditory cortex (denoted “CTX”) [14, 37]. Note that the brainstem responses at latencies < 4 ms were highly consistent in morphology and latency across cats. There was greater variability for the IC, TC, and CTX responses, possibly due to differential effects of habituation for those more central-level generators [45], or variations among cats in the distortion of current paths by the skull cylinder and its acrylic cement.

Figure 3b shows the folded eFFR waveforms recorded across all pulse rates for Cat BR. Both recording channels show periodic structures occurring at the rate of each stimulus and, at least for the ipsilateral channel, these were visible up to the highest pulse rates. Note, that at the higher rates, the CN (see Ipsi Channel) increases in latency, from 1.2 to 1.4 ms, and reduces in amplitude, likely due to habituation at the high pulse rates. The individual peaks that were attributed to various putative generators in Fig. 3a are most apparent at pulse rates below ~ 100 pps. Those separate responses begin to overlap with each other at higher pulse rates having shorter inter-pulse intervals; for instance, the short-latency responses to later pulses progressively shift to earlier time points and superimpose on the broader long-latency responses to earlier pulses. That observation is consistent with the eFFR at medium and high pulse rates reflecting the summed stimulus-synchronized activity of multiple neural generators.

The eFFR waveforms in Fig. 3a and b also revealed a strong influence of recording-electrode laterality on the recordings. The largest amplitudes in the ipsilateral channel tended to occur for the short-latency CN and SOC responses, which were also stronger than those recorded in the contralateral channel. Those short-latency brainstem responses remain visible across nearly all pulse rates, whereas they are relatively weak in the contralateral channel and become less distinct with increasing pulse rate. That asymmetry likely reflects the closer proximity of ipsilateral active electrode to the stimulated auditory nerve and cochlear nucleus. Within the contralateral channel, the largest amplitudes tended to occur for the longer-latency IC and later responses. Indeed, only the contralateral channel consistently showed a distinct IC peak across the eFFR recordings (see Fig. 3a), likely because the contralateral active electrode was more proximal to the primary midbrain projections to the contralateral auditory pathway. Those hemispheric asymmetries aided the interpretation of between-channel differences in the remaining eFFR results.

### eFFR Amplitude and Phase Analyses

**Fig. 4:**
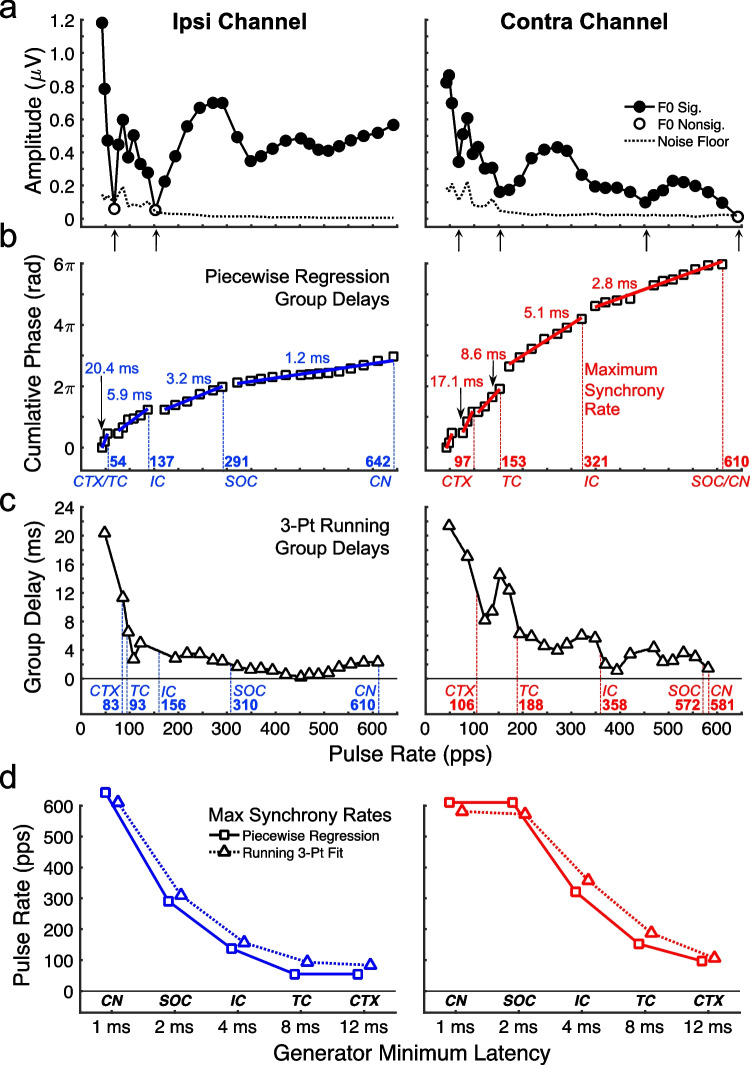
Example of the eFFR analysis for Cat AB on post-implantation week 10. Ipsi and contra are the hemispheres of the recording electrodes with respect to the hemisphere of the CI. **a** Amplitude transfer functions: Filled and open symbols indicate responses that respectively were or were not significantly phase locked to the pulse trains. Upward arrows below the horizontal axis indicate prominent dips in amplitude at pulses rates that were omitted from corresponding phase transfer functions (Fig. 4b). Dotted lines in Fig. 4a show the noise level estimated at each pulse rate from the spectral bins adjacent to the F0. **b** Cumulative phase transfer functions with the solid line segments depicting results of the piecewise regression method for measuring group delays. Group delays in milliseconds are labelled next to each segment of the regression line. **c** 3-pt running group delays computed for each consecutive set of three pulse rates (symbols) with linear interpolation between pulse rates (solid line). Vertical dashed lines in **b** and **c** indicates the highest pulse rate (i.e., maximum synchrony rate) that could be attributed to each putative neural generator based on the group-delay estimated latencies (see text for details). Generators are labelled along the horizontal axes with the corresponding maximum synchrony rates given in pps. **d** Maximum synchrony rates in this example cat extracted by piecewise regression (squares) and 3-pt running group delays (triangles) for each of the neural generators. Generators in b-d are denoted by CN, cochlear nucleus; SOC, superior olivary complex and other brainstem structures; IC, inferior colliculus; TC, thalamocortical; CTX, cortex

The analyses of eFFR amplitude and phase transfer functions are demonstrated for Cat AB in Fig. 4a–d; left and right panels depict ipsi- and contralateral recording sites. As exemplified in Fig. 4a, the eFFR amplitudes were generally largest at the lowest pulse rates and decreased in a non-monotonic manner at the higher pulse rates. Amplitudes at the highest rates tended to trend upward in the ipsi- but not the contralateral channel, reflecting the persistent influence of the CN response on the side of the stimulated auditory nerve. Importantly, the patterns of amplitudes across rates were characterized by multiple peaks and dips, resembling the spectral amplitude features of normal-hearing FFR that have been attributed to the frequency-specific interactions of multiple neural generators measured simultaneously at the scalp [33]. Whereas we often observed significant eFFR values (filled markers) even at the highest tested pulse rates, non-significant responses (open markers) often corresponded with pronounced dips in the amplitude (upward arrows), presumably because of destructive phase interference at those pulse rates. As a result, pulse rates corresponding to those dips and any other non-significant responses could yield ill-defined measures of phase lag and were omitted from the phase analysis described below.

Figure 4b shows the unwrapped cumulative eFFR phase lags that increased monotonically from low to high pulse rates. The group delays computed over those progressions of phase provided estimates of the eFFR response latency. As shown by this example, we observed systematic transitions in the cumulative phase from steeper slopes (i.e., longer group delays, values given in ms) at low stimulus pulse rates to shallower slopes (i.e., shorter group delays) at higher rates. Similar transitions have previously been reported in FFR of normal-hearing animals [25] and suggests that the phase responses are dominated by higher-level auditory generators (e.g., cortical) at low rates and lower-level generators (e.g., brainstem) at higher rates. We examined those latency transitions using the piecewise linear regression and 3-pt running fitting methods (see the “Methods” section for details).

The piecewise regression method quantified up to five discrete latency transitions, as shown by the blue and red fitted line segments in Fig. 4b. The resulting group delays tended to show an orderly pattern of latencies that could be related to the putative generators. Namely, the lowest pulse rates were characterized by latencies appropriate to cortical responses (> 12 ms), followed at slightly higher rates by thalamocortical latencies (8–12 ms). Those generator latencies tended to coincide with the relatively large eFFR amplitudes (Fig. 4a) at the lower pulse rates. The intermediate pulse rates showed latencies corresponding to the inferior colliculus (4–8 ms) or brainstem (2–4 ms) generators, whereas the highest rates tended to show only latencies relevant to the CN (1–2 ms; see Ipsi Channel). Note, the best-fitting lines tended to segment between pulse rates showing large dips in amplitude, which typically coincided with a marked transition in group delay (see the Contra Channel line segments of 8.6 ms vs. 5.1 ms).

The 3-point running group delay method is shown in Fig. 4c and provided a more continuous estimate of response latency as a function of pulse rate. Group delays between the measured pulse rates were inferred by linear interpolation in 1-pps steps. As in the piecewise regression method, the 3-pt group delays were consistent with cortical and thalamocortical generators at the lowest pulse rates, inferior colliculus or brainstem generators at the intermediate rates, and only the CN at the highest rate. The running group-delay estimates were more vulnerable than the piecewise regression method to local deflections in the cumulative phase, perhaps due to noise or effects of phase interference, and were therefore sometimes non-monotonic with increasing pulse rate. That effect could cause relatively large swings in group delays at low pulse rates (see Contra Channel at < 200 pps) and at high rates could cause instances in which short-latency group delays dropped below 1 ms (see Ipsi Channel at ~ 450 ms). For that reason, as well as to avoid possible influence by any residual electric artifact, pulse-rate segments having group delays < 1 ms were omitted from further analysis.

### Maximum Synchrony Rates of Neural Generators

The estimates of eFFR latency in Fig. 4c and d provided a correlate of the phase-locking capacities of neural generators along the ascending auditory pathway. The so-called maximum synchrony rate for a given generator was the highest pulse rate having a group delay as long or longer than the minimum latency attributed to that generator. For example, the earliest latency attributed to an inferior-colliculus response was 4 ms (described for Fig. 3a). In each panel of Fig. 3c and d, the vertical dashed lines that extend towards the “IC” labels along the horizontal axes mark the highest pulse rates having a delay of ≥ 4 ms. The corresponding rate values are given in pps and were interpreted as the highest rate at which an IC generator could dominate the eFFR within a given recording. Within each recording channel, the two methods of group delay analysis gave comparable IC maximum rate values, including 137 to 156 pps in the ipsilateral and 321 to 358 pps in the contralateral channel. Note that higher maximum rate values for the contra- than the ipsilateral channel do not necessarily indicate that IC generators phase locked to higher pulse rates in the contralateral hemisphere. Rather, latency estimates in the contralateral channel were less influenced by short-latency brainstem responses (CN and SOC), which were more prominently recorded in the ipsilateral channel (see Fig. 3) and could thereby skew the ipsilateral group delays towards lower latencies than was measured contralaterally. Therefore, in this example, 358 pps was taken as the highest measurable pulse rate at which phase locking of the IC generators could dominate the phase response.

In that way, we also quantified the maximum synchrony rates for each of the putative generators based on the nominal minimum latencies of 1 ms (CN), 2 ms (SOC), 4 ms (IC), 8 ms (TC), and 12 ms (CTX), each marked by vertical dashed lines in Fig. 4b and c. Figure 4d plots those maximum synchrony rates for the example cat AB, estimated using piecewise regression (squares; corresponding to Fig. 4b) and 3-point running group delays (triangles; corresponding to Fig. 4c). The maximum synchrony rates measured across cats and recording channels will be considered in the remaining results.

### Test–Retest Reliability and a Delay-and-Add Model of the eFFR

**Fig. 5:**
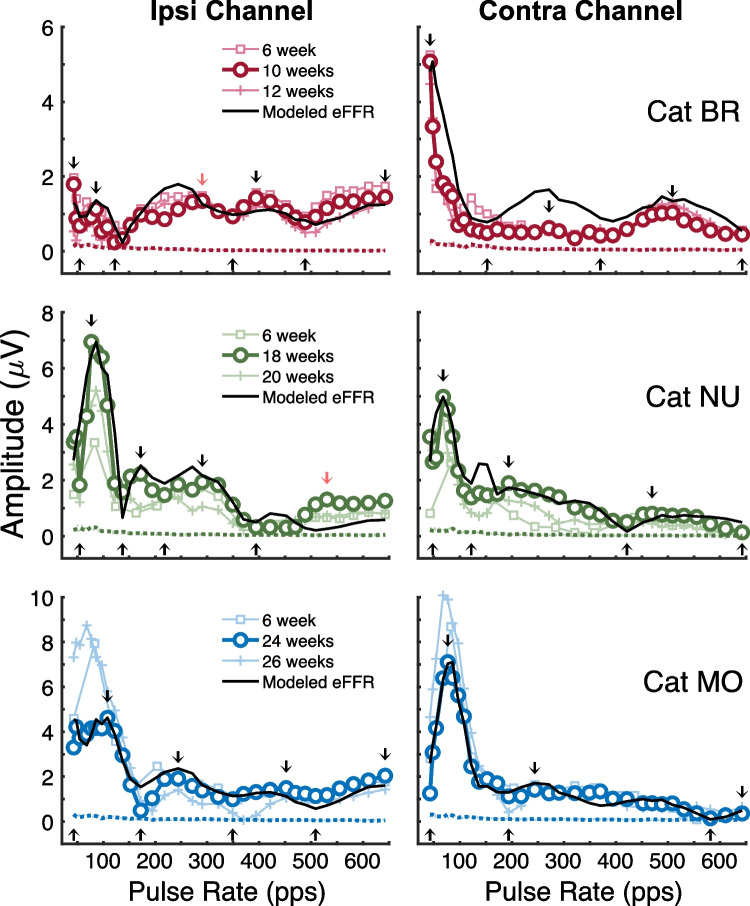
eFFR amplitude transfer functions for three recording sessions in three cats. The thick lines and symbols are the representative sessions of weeks ~10, 18, and 24 for cats BR, NU, and MO, respectively. Lighter thin lines are recording sessions at ~6 weeks after implantation (square markers) and ~2 weeks after the representative session (12, 20, and 26 weeks; + markers). Black lines show the best-fitting delay-and-add model of the eFFR amplitudes for the representative session. Arrows depict the peaks (down arrow) and dips (up arrows) selected for each recording that were used to fit the model

**Fig. 6:**
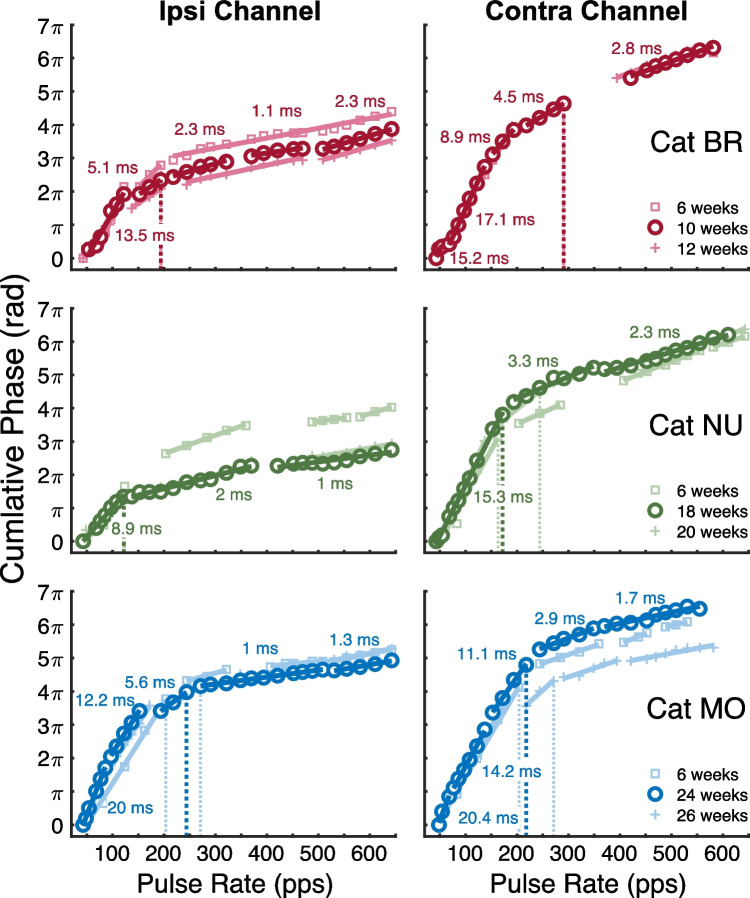
eFFR cumulative phase transfer functions for three cats (same as Fig. 5) shown for three experimental sessions. The piecewise regression applied to each recording is shown by straight lines with corresponding group delays indicated only for the representative recording session. The dashed vertical line indicates the highest pulse rate corresponding to group delays that could be attributed to the IC generator (i.e., ≥4 ms)

Figure 5 shows the individual amplitude transfer function for the ~ 10-, 18-, and 24-week representative recording session in three cats; the corresponding phase transfer functions are shown in Fig. 6. In addition, data are shown from 6 weeks after implantation and 2 weeks after the representative session for each cat. The figure demonstrates the wide variation in amplitude patterns that were observed across individual cats and between the two recording channels. Nevertheless, the inter-session amplitude patterns for each cat and channel were highly replicable, such that peaks (down arrows) and dips (up arrows) tended to occur at similar pulse rates across recordings. Across the examples in Fig. 5, there was an 86.4% hit rate (38/44) in the correspondence of peaks and dips between the representative sessions and those of the other sessions, measured within each cat and recording channel. A hit was counted when peaks or dips coincided within ± 32 pps between two sessions, which was the largest increment in the tested pulse rates, whereas a miss was the nearest corresponding peak or dip that was > 32 pps. By contrast, when the same comparison was made between sessions of different cats, randomly selected with replacement over 200 permutations, the 95th percentile of hit rates was 68.2% (22/44). Inter-session variability, however, was sometimes apparent in the overall amplitudes at the lower pulse rates (< 200 pps; see cats NU and MO). Those amplitude differences were possibly due to thalamocortical or cortical responses at those lower pulse rates showing greater sensitivity to nuisance recording variables (e.g., electrode placement) or possibly sedative levels across sessions.

Next, we examined whether the delay-and-add model of multiple neural generators (described in Methods) could accurately reproduce each cat’s patterns of peaks and dips in eFFR amplitude. The model (Fig. 5, black lines) generally performed well in capturing the overall amplitude profiles of individual cats and recording channels. Across all examples in Fig. 5, there was a 95.5% (42/44) hit rate in the correspondence between the empirical peaks and dips of the representative eFFR and those of the best-fitting models (misses are indicated by red arrows). Among all hits and misses, the magnitudes of pulse-rate errors had a median of 0 pps (25th–75th quartiles = 0–13.2 pps). The overall amplitude difference between each empirical and modeled transfer function was computed by the RMS of the amplitude errors across pulse rates, which had a median of 3.4 dB (25th–75th quartiles = 3.0–5.7 dB); that corresponds to a factor of 1.58 (25th–75th quartiles = 1.41–1.93). Of the four generator sinusoids (ordered: CN, SOC, IC, and TC/CTX), the CN had a fixed 1.2-ms delay latency, whereas the longer generators were allowed to vary within their respective latency ranges and had median best-fitting latencies of 3.7, 5.2, and 13.6 ms. The magnitude scalars applied to the four generators had medians of 1, 1, 2, and 8.5, respectively; here, only the IC and TC/CTX generators were allowed to vary within limited ranges of scalars. That excludes Cat BR’s ipsilateral recording, which showed relatively small amplitudes at low pulse rates and could only be modeled by reducing the magnitude of the longer-latency generators and increasing the CN magnitude (scalars = 3, 1, 2, 2).

To evaluate the significant contribution of each generator in the model performance, we removed one generator at a time and refit the model to the empirical amplitudes. Each of those 3-generator models were compared to the full 4-generator model based on (1) the peak and dip correspondence (hit rates and magnitudes of pulse-rate errors) and (2) the RMS-amplitude errors, as assessed by Wilcoxon signed rank tests (*p* < 0.05). By those measures, the model fits were significantly poorer when removing any one of the generators. That is, compared to the full model, when removing CN, SOC, IC, and TC/CTX, hit rates dropped from 95.5 to 68.2, 72.7, 65.9, and 72.7%, respectively (*p* = 0.043, 0.028, 0.043, 0.028), median magnitudes of pulse-rate errors increased from 0 pps to 19.0, 11.5, 16.5, and 20.0 pps, respectively (*p* = 0.0014, 0.0017, 0.00049, 0.00041), and median RMS-amplitude errors increased from 3.4 to 5.0, 4.9, 5.8, and 8.3 dB (*p* = 0.046, 0.046, 0.12, 0.12); note, models omitting the IC or TC/CTX generators produced the largest RMS errors but were non-significantly different from the full model, which was likely because of the small sample size (*n* = 6). Finally, to assess the role of simulating the reduced phase-locking capacities of high-level auditory nuclei, we removed the high-frequency taper of the IC and TC/CTX generators, which in the original model were linearly reduced to zero amplitude starting at 250 and 90 Hz, respectively. Thus, with the taper removed, the IC and TC/CTX generators had equal influence across all pulse rates. Those changes had slightly less impact on the overall hit rate of the model but still produced significantly worse pulse-rate and RMS-amplitude errors compared to the original model. For removing IC and TC/CTX cutoffs, respectively: hit rates = 86.4 and 84.1% (*p* = 0.23, 0.068); median pulse-rate errors = 19.8 and 7.0 pps (*p* = 0.002, 0.019); and median RMS-amplitude errors = 11.6 and 7.4 dB (*p* = 0.028, 0.028). Together, these results suggest that each of the sinusoidal generators and their latency and cut-off parameters were necessary to the 4-generator model performance in Fig. 5.

**Fig. 7:**
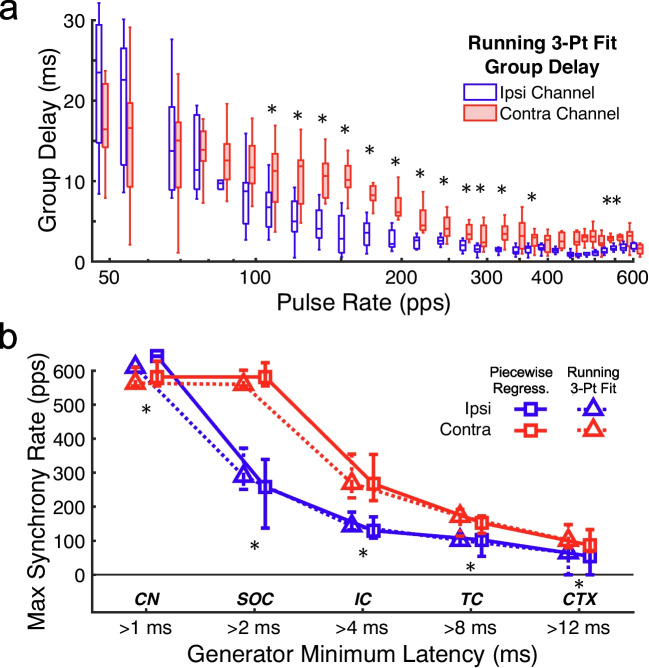
**a** Boxplot distributions of 3-pt running group delays for all cats, showing the median, 25th and 75th quartiles, and minimum and maximum values (whiskers). Red and blue colors indicate the ipsi- and contralateral channel, respectively. **b** Maximum synchrony rates depicted for each putative neural generator (horizontal axis), comparing both group delay measures (piecewise regression and running 3-pt fit) and each recording channel. Symbols with error bars indicate the median and 25th and 75th quartiles across cats. Asterisks in **a** and **b** denote significant pairwise differences assessed by the Wilcoxon signed rank test, with 5% FDR corrections for multiple comparisons

Figure 6 shows the piecewise regression analysis for the individual examples of cumulative phase with group delays given only for the representative recording session. These plots illustrate two important observations. First, as with the eFFR amplitude, the cumulative phase patterns were somewhat distinct across cats and recording channels. Nevertheless, the group delays at a given pulse rate and the break points of the fitted segments were mostly consistent across multiple recording sessions from individual animals. For example, the dashed vertical lines show the maximum synchrony rate of the putative IC generator, defined as the highest pulse rate for line segments with a group delay of ≥ 4 ms. We computed the pulse-rate differences of IC maximum synchrony rates between sessions for each cat in the study (*n* = 9; excluding cat CL, see the “Methods” section) and each recording channel. The magnitude of inter-session differences had a combined median value of 20.8 pps and ranged from 0 to 138.1 pps; (interquartile range = 77.5 pps); for reference, the median increment in the tested stimulus rates was 20.7 pps. Second, the contralateral channel frequently showed longer group delays at the intermediate-to-higher pulse rates than the ipsilateral channel, which is evident in the present example by the steeper phase progressions and larger maximum cumulative phase in the contralateral than the ipsilateral channel. Consistent with that observation, the IC maximum synchrony rates were highest in the contralateral channel in two of the three example cats; that laterality effect is considered below in Fig. 7.

Of the examples shown in Figs. 5 and 6, the 6-week recording sessions of cats NU and MO utilized only 19 of the 31 stimulus rates tested in the representative sessions, evident by the coarser spacing of square markers for those cats. Importantly, neither cat showed systematic changes in amplitudes nor patterns of cumulative phase despite their representative recording occurring at later post-implantation durations (i.e., ≥ 18 weeks) than the other cats (i.e., ~ 10 weeks). Note that the vertical offsets of phase segments seen between some of the recording sessions (e.g., MO weeks 24 vs. 26) could result as a byproduct of single-point variations in the phase unwrapping process; for instance, when a phase difference between a given pair of rates was approximately π (i.e., falling just below or above), then 2π could be added to the cumulative phase values for one recording but not another. Nevertheless, the slopes of phase segments and the transitions in group delays remained largely stable across sessions.

### Comparisons of eFFR Recording Hemispheres

Group delays measured for all 10 cats formed the basis for comparisons between recording hemispheres. The distributions of the group delays in Fig. 7a were given by the 3-point running group delay method, which, unlike the piecewise regression method, provided a rate-by-rate comparison of group delays between channels. For pulse rates below ~ 80 pps, both channels show median group-delay values of 11.4 to 23.5 ms, indicative of thalamocortical or higher-level generators. That the distributions were particular wide at those lower rates was likely related to the large between-cat variability generally observed for cortical responses, as previously noted for Fig. 3a, or local deflections in the cumulative phase that differed across cats, which could cause swings in the running group delays at low rates as noted for Fig. 4c. At pulse rates > 80 pps, group delays in both channels gradually decreased, but the median values of the contralateral channel (red boxes) remained mostly higher than those of the ipsilateral channel (blue boxes). Those results were reflected by significant main effects of pulse rate (2-way Rate × Channel RMANOVA, F(28,252) = 20.65, *p* < 0.001) and recording channel (F(1,9) = 28.09, *p* < 0.001). The interaction of those factors was not significant after the Greenhouse–Geisser correction (*p* = 0.087). Pairwise comparisons show that the contralateral channel contained significantly longer group delays than the ipsilateral channels predominately for the intermediate pulse rates of ~ 110 to ~ 370 pps (Wilcoxon signed rank, *p* = 0.0176–0.0020; FDR corrected). Those pairwise differences were possibly related to greater midbrain and thalamocortical synchrony recorded in the contralateral hemisphere; namely, at the significantly differing pulse rates below 370 pps, median group delays for the contralateral channel had 25th to 75th quartile ranges of 3.4 to 10.4 ms, compared to the shorter brainstem latencies represented in the ipsilateral channel ranging from 1.9 to 3.9 ms over the same rates.

The group-delay differences between recording channels suggest that the ipsilateral channel was more influenced by the short-latency CN or SOC generators proximal to that recording electrode. By contrast, the contralateral recordings might better reflect the upper limits of neural synchrony by longer-latency IC and higher-level generators, namely because those longer-latency responses showed less competition from CN and SOC generators in the contra- than in the ipsilateral channel. We tested that hypothesis by comparing the maximum synchrony rates for each of the putative generators (Fig. 7b); the derivation of the maximum synchrony rates was demonstrated for one example cat in Fig. 4b–d. We began by testing whether the piecewise regression and 3-point running group delays yielded similar maximum-synchrony results, which was confirmed by a repeated-measures analysis showing no significant main effect of phase-lag fitting method (3-way RMANOVA Generator x Fitting Method x Channel, F(1,9) = 0.18, *p* = 0.68). As expected, there was a significant main effect of generator, so that the synchrony of longer-latency generators was limited to lower stimulus rates (F(4,36) = 303.45, *p* < 0.001). That generator effect also showed a significant interaction with recording channel (F(4,36) = 38.12, *p* < 0.001, G–G corrected). That interaction effect was also observed when a 2-way model included only the piecewise regression (Generator x Channel, F(4,36) = 15.75, *p* < 0.001, G–G corrected) or 3-point running group delay (F(4,36) = 31.95, *p* < 0.001, G–G corrected) methods. The interaction was due to the contralateral channel showing higher maximum synchrony rates for all generators except the shortest-latency CN response, which in a pairwise comparison had significantly higher synchrony rates in the ipsilateral channel (Wilcoxon signed rank, *p* = 0.0078, FDR corrected). Note, pairwise comparisons for each generator used group-delay values averaged across the two fitting methods. Conversely, maximum synchrony rates were significantly higher in the contralateral channel for the SOC, IC, TC, and CTX generators (Wilcoxon signed rank, *p* = 0.0039–0.0020, FDR corrected). Together, those maximum pulse-rate estimates suggest that neural synchrony in the cat is measurable by the eFFR for the SOC, IC, TC, and CTX generators up to respective median rates of ~ 570 pps, ~ 268 pps, ~ 162 pps, and ~ 94 pps.

## Discussion

The present study evaluated the eFFR elicited by constant-amplitude electrical pulse trains in the deafened cat model of chronic CI implantation. We showed that the eFFR is detectable, distinct from the electric artifact, across a broad range of stimulation rates (43 to 642 pps) and differs systematically in amplitude and phase-lag patterns according to the ipsi- or contralateral recording configuration. Based on the amplitude and phase transfer functions, we adapted the approaches of delay-and-add modeling and phase-lag segmentation, which provided strong evidence for multiple neural generators of the cat eFFR, commensurate with its normal-hearing counterpart. Finally, we utilized group-delay estimates of eFFR latency to derive estimates of the maximum pulse rates of phase locked activity by putative generators from the auditory brainstem to cortex. Here, we discuss (1) some practical considerations regarding eFFR detection from electric artifact, (2) the origins of the eFFR from multiple generators, (3) the maximum synchrony rates as compared to other animal studies, and (4) implications for non-invasive studies of temporal processing with the CI and novel auditory prostheses.

### Isolating eFFR from Artifact and Noise: Cat vs. Human

A major challenge for measuring stimulus-synchronized electrophysiological responses to electrical stimulation is the separation of neural activity from the overlapping artifact. Among the techniques that have been developed to remove electric artifact (reviewed by [46]), the blanking method is computationally simple, can be applied to single recording channels (as opposed to, e.g., beamforming techniques that require multiple channels), and requires few assumptions about the artifact composition. Blanking has been most successful in measurements of auditory steady-state responses (ASSR) evoked by the envelopes of amplitude-modulated pulse trains [47–50]. In that case, blanking is applied to carrier pulse rates (i.e., 500 to 1000 pps) much higher than the envelope-following neural responses (i.e., 1 to 100 Hz). In the case of eFFR for constant-amplitude pulse trains, however, the blanked samples can overlap in time with and potentially distort the neural responses, particularly with blanking lengths exceeding 50% of the inter-pulse interval [28]. Moreover, the artifact by clinical CIs contains radio frequencies that transmit power and data to the CI, as well as extended voltage decays that last several milliseconds after pulse onset due to volume conduction in the human head [31]. Those artifact features have largely restricted human eFFR measures to low pulse rates of < 50 pps [32], although eFFRs were more recently measured as high as ~ 160 pps after applying blanking of up to 3.5 ms [28]; note, that blanking length was 60 times the duration of the 58-µs biphasic-pulse stimuli used in that study.

The template subtraction approach is an alternative that can potentially attenuate the artifact while largely preserving the neural response [29, 30], although its success depends strongly on the accuracy of modeling the response-free artifact. Notably, a recent human CI study by Gransier and colleagues (2024) showed that combining template subtraction—applied primarily to the artifact voltage decay—with blanking of just 0 to ~ 400 µs re pulse onset could improve eFFR detection at higher pulse rates [29]; in this case, the blanking length was just 5 times the biphasic-pulse duration of 80 µs. Nevertheless, neural responses were only detected at a maximum pulse rate of ~ 233 pps, considerably lower than the stimulus rates at which normal-hearing FFR are detected [18]. That result might be explained in part by impaired temporal processing of those CI users, although further studies are needed.

Contrasting with the human studies, the present eFFR in cats demonstrated stimulus-synchronized neural activity over a broad range of stimulus rates, including responses as high as ~ 640 pps. That result highlights some key aspects of scalp recordings in animals that are likely beneficial to both artifact removal and response detection. Those include (1) use of direct percutaneous connection, which obviates the need for radio-frequency power and data transmission, (2) shorter-duration artifacts, as long-lasting voltage decays were not evident, and (3) improved signal-to-noise ratio of neural responses due to subcutaneous electrodes with low impedance (i.e., no skin resistance) and closer proximity to neural generators in the smaller animal heads (see [18]). Here, we found that template subtraction could effectively attenuate the artifact to levels near the neural response. Only a limited blanking length of 800 µs was needed to remove residual errors in pulse-by-pulse template matching, which presumably were due to recording noise; as in Gransier et al. [29], that blanking length was ~ 5 times the duration of 164-µs biphasic pulses used here. Although we could not entirely rule out remaining influences of artifact or byproducts of artifact reduction (e.g., interpolation), we argue that those influences were small relative to the neural responses, which was most evident by group-delay latencies that were attributable to well-defined peak responses in the eFFR waveforms. Moreover, the higher prevalence of short group-delay latencies in the ipsi- than the contralateral channel could not be explained by larger artifacts recorded at ipsilateral hemisphere. That is because the artifact differences between recording channels (before artifact reductions) varied widely across cats, including 4/10 cats that showed 1.2 to 3.6 times larger artifact in the contralateral channel; we attribute that variation to voltage paths around the head-mounted cylinder and its acrylic cement.

### Evaluating Multiple eFFR Generators

That we could detect phase-locked responses often across the entire range of stimulus rates may, at first glance, appear contrary to the impaired temporal acuity of CI users. We could explain that result, however, by the contributions of multiple eFFR generators, whereby phase locking of lower-level generators (i.e., brainstem) persisted across most or all pulse rates but was diminished for the higher-level auditory nuclei (i.e., cortical or midbrain) at higher rates. That interpretation was supported in part by the delay-and-add modeling of the eFFR amplitude transfer functions. Theoretical generators consisted of the sums of four sinusoids, each with biologically plausible delays corresponding to CN, SOC, IC, and TC/CTX, which simulated the ensemble of volume-conducted potentials measured at the scalp. Depending on the F0, the sinusoids had either constructive or destructive phase interactions that could reproduce the frequency-specific amplitude variations of the eFFR recordings (Fig. 5). Our model was most comparable to that by Tichko and Skoe [33] developed for normal-hearing human FFR and which expanded on an earlier brainstem-only version ([24]; normal-hearing cats) to include midbrain and cortical generators as well as relative scaling of generator magnitudes. Like that study, by selectively removing generators, we showed that all generators, from brainstem to cortex, were necessary to achieve robust model performance. Model fits were also significantly improved by simulating the limited phase locking of the long-latency generators to high pulse rates (i.e., tapering the contributions of IC and TC/CTX) so that only the short-latency generators contributed at pulse rates > 250 Hz. Moreover, the model accommodated a wide variety of amplitude profiles across cats, likely because of the magnitude scaling of generators that accounted for variations in generator-to-recording distances and individual cat physiology and voltage paths. A limitation of the model is that its simplifying assumptions (i.e., number of generators, sinusoidal signals) preclude estimations of actual neural response latencies based on the modeled delays. As discussed by Tichko and Skoe [33], those delays are also inherently ambiguous due to the relevant phase interactions arising from the relative (i.e., spacing between delays) rather than absolute delay of each generator. Nevertheless, the delay-and-add model lends proof-of-principle evidence of the multiple-generator activities in the cat eFFR.

More direct evidence of multiple generators was given by estimates of the eFFR latency derived from the phase transfer functions. The progressions of group delay were quantified by two methods of phase slope analysis, which indicated that the relative contributions of generators varied with pulse rate, from thalamic or cortical latencies (> 8 ms) at low rates to midbrain or brainstem latencies at mid-to-high rates. That pattern is generally consistent with the declining upper limits of phase locking in the ascending auditory pathway [51, 52]. Latency transitions of stimulus-synchronized scalp recordings have been previously reported by normal-hearing animal and human studies (e.g., [39, 49, 53]). For CI stimulation, one study of acutely implanted guinea pigs reported a bisection of ASSR group delays in response to amplitude-modulated electrical sinusoids (22.1 ms at < 55 Hz, 2.2 ms at > 65 Hz) [54]. The presence of multiple latency transitions reported here, however, was more comparable to ASSR recordings in normal-hearing rabbits, which showed cortical delays of ~ > 20 ms at low modulation frequencies of < 80 Hz, midbrain delays of ~ 5 ms at < 300 Hz, and brainstem delays of ~ 3 ms at > 300 Hz [25]. Notably, the group delays in that study corresponded well with near-field neural delays recorded in the same animal’s auditory cortex, inferior colliculus, and superior olivary complex, respectively. Moreover, the dominance of one generator among other ongoing sources was tested causally by suppressing the cortex with topically applied potassium chloride, which left only midbrain-like delays at the lowest modulation frequencies.

### Maximum Synchrony Rates of Normal- and Electric-Hearing Cats

Based on the group-delay latencies, we inferred the upper limits of phase locking to electric TFS at various stages of the auditory pathway. Those maximum synchrony rates were the highest pulse rates at which each generator could be judged to dominate the eFFR by yielding group-delay latencies attributed to that generator. The minimum latency of each generator was in turn determined by the peak eFFR waveform responses examined at low stimulus rates (Fig. 3) and whenever possible was corroborated by published measures of neuronal spiking delays by electric stimulation. Take for example the minimum latency of 4 ms for the IC generator. The eFFR waveforms peaks in Fig. 3 that were attributed to the IC response had a median among cats of 5.3 ms and ranged from 4.0 to 6.5 ms (*n* = 10, 25th–75th quartiles = 4.8–6.0 ms). That range overlaps with Middlebrooks and Snyder’s [16] measures of first-spike latencies of inferior-colliculus neurons in acutely implanted cats, which had a median of 6.7 ms, ranging from 4.0 to 9.1 ms (*n* = 100; 25th–75th quartiles = 6.2–7.4 ms). That the eFFR latencies were slightly earlier than the spiking activity can be explained by the higher current levels tested here—Middlebrooks and Snyder tested levels only 4 dB above unit threshold—as well as the likelihood that far-field potentials also reflect input activities to the generators (e.g., excitatory postsynaptic potentials) in addition to the spiking outputs [55] (see also [24, 25]).

We regard the maximum synchrony rates as positive evidence that a generator can phase lock at least as high that pulse rate. We are careful, however, not to suggest that we have identified the generator’s absolute limit of phase locking, as can be obtained by single- and multi-unit recordings from isolated auditory nuclei. That is because maximum synchrony rates depended on the relative dominance of generators in the recorded waveforms. For instance, a longer-latency generator might contain a population of neurons that phase lock at a higher stimulus rate than is evident in the eFFR, but the contribution of that population to the eFFR at those rates could be obscured by the influence of higher-amplitude shorter-latency responses. That was evident from the comparison of the two recording channels (see Fig. 7), wherein the brainstem, inferior colliculus, thalamocortical, and cortical generators each had higher maximum synchrony rates in the contra- than the ipsilateral recording channel. That does not mean that phase locking of those generators was reduced within the ipsilateral brain structures, but rather that their FFR signatures in the ipsilateral channel were relatively weak compared to responses by the shorter-latency cochlear nucleus, recorded more proximally by that electrode. Therefore, the maximum synchrony rates of a generator should be regarded as the highest discernable value based on the experimental conditions. In the following, we focus on results from the contralateral recordings, which emphasized the maximum phase locking by the longer-latency generators.

We were particularly interested in maximum synchrony rates at generator levels of the inferior colliculus or higher, which have potentially greater relevance to perception and have received extensive study of synchronous spiking activity by electric stimulation (e.g., [10–12, 15, 16, 56]). Importantly, the inferior colliculus can preserve synchronized or isomorphic coding of periodicity at relatively high stimulus rates, whereas non-isomorphic transformations are thought to occur at the thalamic and cortical levels, including non-synchronized rate or neural place coding [51, 57]. For instance, in normal-hearing cats, the upper limit of phase-locked spiking by cortical neurons rarely exceeds 50 sine-wave or click cycles per second (e.g., [58, 59]) but has been measured in the inferior colliculus as high as 600 per second [51, 60, 61]. Note that normal-hearing measures of the upper phase-locking limit at high acoustic pulse rates are constrained by effects of cochlear filtering (e.g., resolved harmonics). In our recent study of the normal-hearing cat, we used similar sedation and scalp-recording procedures as the present study and showed that FFRs were elicited by spectrally unresolved acoustic pulse trains resembling those by a CI electrode positioned in the basal cochlea [20]. Different from the present study, the normal-hearing FFR did not exhibit thalamic- or cortical-like group delays at lower pulse rates, although the lowest tested acoustic rate was only 94 pps, a rate at which at least cortical group delays in the present eFFR were highly diminished. That result might also have been due to cortical habituation over the 12-s-long acoustic pulse trains used in that study (compared to 500 ms here) or possibly to other differences between acoustic vs. electric activation of the auditory pathway. Nevertheless, midbrain-like group delays of ~ 5.3 ms were consistently obtained with the normal-hearing FFR up to 640 pps. By contrast, the maximum synchrony rates of the inferior-colliculus generator in the present study had an estimated median pulse rate of ~ 268 pps. Those comparisons to normal-hearing cat studies would tentatively suggest that inferior-colliculus phase locking in the CI-implanted cats was reduced from that of normal hearing, either because of a property of the electrical stimulation per se or because of some degradation of the auditory pathway due to deafening followed by only intermittent stimulation after implantation.

Short-term studies in anesthetized cats have characterized the phase-locking capacities of neurons in the inferior colliculus*.* Single- and multi-unit spiking activity was recorded in response to electric cochlear stimulation at varying electrical pulse rates. A common finding among those studies was a boundary of ~ 300 pps above which inferior-colliculus neurons no longer synchronize to the stimulus. For example, a set of studies employing bipolar stimulation by pairs of intracochlear electrodes found that groups of adult-deafened cats, most resembling the present study, had average maximum phase-locking rates across neurons of ~ 100 pps and maxima by any neuron of ~ 340 pps [10, 36, 56]. Our previous study of acutely deafened cats, which used CI arrays and monopolar stimulation more like the present study, showed median limiting rates of 220 pps with only ~ 18% of neurons phase locked at a pulse rate of 300 pps [16]. Those values are comparable to the present eFFR estimates of inferior-colliculus maximum synchrony rates eFFR (median of 268 pps) ranging from 189 to 352 pps among cats. The scalp-recorded maximum synchrony rate, therefore, provides a promising alternative to single- and multi-unit recordings for studying the neural transmission of electric TFS at the level of the auditory midbrain.

Previous studies in animals have also demonstrated marked effects of neurodegeneration on central auditory phase locking following deafness [9, 10, 13, 62]. It was beyond the scope of this study to examine such deafness-induced neural changes, and we instead focused primarily on single recording sessions for each cat at ~ 10-weeks post-implantation (~ 12-weeks post-deafening); two exceptions were cats NU and MO with reported data at ≥ 18 weeks post-implantation. Nevertheless, we observed that idiosyncratic patterns of amplitude and phase responses by individual cats were highly reproducible across three selected recordings that were examined for test–retest reliability (Figs. 5 and 6). Notably, that included eFFR comparisons for cat NU and MO from week 6 to weeks respectively ~ 20 and 26 post-implantation. Whereas previous studies of neurodegeneration have left animals completely unstimulated after deafening, we speculate that the within-cat consistency of eFFR in this study was related to the ~ 1–2 h of stimulation every ~ 2 weeks. That is, intermittent stimulation beginning 4 weeks after deafening was sufficient to preserve temporal responsiveness over time, including the maximum synchrony rates of the inferior-colliculus generator (Fig. 6). Future studies can use the eFFR measures developed here for more detailed investigations of central auditory changes related to deafness variables (e.g., duration, auditory-nerve fiber loss) and to test the hypothesis that intermittent stimulation is sufficient to ward off continuous neural degeneration of the central pathways as opposed to complete auditory deprivation.

### Implications for Auditory Prosthesis Research

The eFFR measures developed here in chronically implanted cats provide a valuable tool to study and potentially address the neural bases of limited temporal acuity by electric stimulation. First, the non-invasive eFFR recordings can serve to relate neural limitations identified by short-term invasive studies to the awake animal’s perception in psychophysical tasks. One such neural limitation was identified by the Middlebrooks and Snyder [16] study of inferior-colliculus neural spiking, which suggested that most present-day CIs, positioned in the middle-to-basal cochlear turns, primarily activate tonotopically high-frequency brainstem pathways that exhibit relatively poor transmission of TFS by electric stimulation, namely, the ~ 300-pps upper phase-locking boundary (described above). In our previous study, normal-hearing cats were trained to discriminate the rates of spectrally unresolved, bandpass-filtered, pulse trains centered around 8 kHz to approximate the same basal cochlear place stimulated by the present CI electrodes [20]. Cats reliably detected increases in pulse rate from base rates of 188 to 658 pps, but performance was degraded for lower (94 pps) and higher base rates (752 pps). Those psychophysical results may reflect the cat’s perceptual sensitivity to temporal pitch, for which the function relating rate discrimination to stimulus rate was shifted upward by ~ 1 octave from that of normal-hearing human discrimination of similar bandlimited acoustic pulse trains [18, 63]. Compared to that normal-hearing baseline, psychophysical measures in chronically implanted cats can help determine whether the limitations on inferior-colliculus phase locking by basal cochlear stimulation—assessed by the eFFR—can account for degraded temporal pitch discrimination at the same pulse rates, or, alternatively, whether the cat’s perceptual sensitivity corresponds better with maximum synchrony rates of other putative neural generators.

Second, the eFFR can be used to evaluate novel modes of auditory prosthesis that might enhance transmission of electric TFS, but which are not yet feasible in humans. In the study by Middlebrooks and Snyder [16], it was found that stimulation by a penetrating electrode implanted directly in the auditory nerve more than doubled the percentage of inferior-colliculus neurons that phase locked at stimulus rates > 300 pps compared to CI stimulation in the same animals. Unlike conventional CIs, the intraneural electrode could selectively activate low-frequency auditory pathways from the cochlear apex which apparently are specialized for high-temporal-acuity transmission of TFS. Based on those results in acutely implanted cats, we are now using the eFFR in conjunction with psychophysics to test enhanced temporal acuity in cats chronically implanted with an intraneural electrode. In the same way, the non-invasive measures developed here can evaluate other novel forms of auditory prosthesis. For example, optogenetic stimulation uses light-emitting sources implanted in the cochlea to activate photosensitive proteins (e.g., opsins) expressed in the spiral ganglion cells [64]. Outstanding challenges of optogenetic stimulation for conveying fine temporal information include the relatively slow channel kinetics of opsins, presently having the shortest time constants of 1–3 ms, as well as strong adaptation by spiral ganglion neurons, which limit light-synchronized responses of those neurons to stimulus rates of < 300 Hz [65]. In the ongoing efforts to optimize both opsin channel efficiency and stimulus coding strategies, the present FFR measures can assess temporal transmission in the central auditory system elicited by optical stimulation—an advantage of which is the absence of electrical artifact.

Finally, the non-invasive eFFR is well-suited for animal studies of long-term factors that can potentially modify temporal acuity by electric stimulation but that are not well controlled in human patients. For instance, studies of long-term deafened cats have shown that restoring auditory-nerve activity with chronic electric stimulation could partially reverse deafness-induced degeneration of phase locking at the level of the inferior colliculus [10, 12, 36]. Those results suggest that stimulus-dependent neuroplasticity plays an important role in restoring temporal acuity in the auditory pathway. Unlike the short-term invasive studies, however, non-invasive studies can address the detailed time courses and potentially the neural loci of those neuroplastic changes over naturalist time periods in which duration of deafness and restoration of auditory stimulation are experimentally determined. Similarly, the eFFR can monitor long-term effects on central auditory temporal processing induced by novel therapeutic interventions, such as neurotropic factors intended to promote spiral ganglion survival and regeneration of neurites towards the intracochlear electrodes [66].

## Data Availability

Data will be made available upon reasonable request from the corresponding author.

## Code Availability

Code that was used in this study will be made available upon reasonable request from the corresponding author.
